# Advances in engineering CRISPR-Cas9 as a molecular Swiss Army knife

**DOI:** 10.1093/synbio/ysaa021

**Published:** 2020-10-24

**Authors:** Grace A Meaker, Emma J Hair, Thomas E Gorochowski

**Affiliations:** 1 School of Biological Sciences, University of Bristol, Bristol BS8 1TQ, UK; 2 School of Biosciences, Cardiff University, Cardiff CF10 3AT, UK; 3 BrisSynBio, University of Bristol, Bristol BS8 1TQ, UK

**Keywords:** synthetic biology, CRISPR, genome editing, ethics, Cas9

## Abstract

The RNA-guided endonuclease system CRISPR-Cas9 has been extensively modified since its discovery, allowing its capabilities to extend far beyond double-stranded cleavage to high fidelity insertions, deletions and single base edits. Such innovations have been possible due to the modular architecture of CRISPR-Cas9 and the robustness of its component parts to modifications and the fusion of new functional elements. Here, we review the broad toolkit of CRISPR-Cas9-based systems now available for diverse genome-editing tasks. We provide an overview of their core molecular structure and mechanism and distil the design principles used to engineer their diverse functionalities. We end by looking beyond the biochemistry and toward the societal and ethical challenges that these CRISPR-Cas9 systems face if their transformative capabilities are to be deployed in a safe and acceptable manner.

## 1. Introduction 

**Figure 1.**
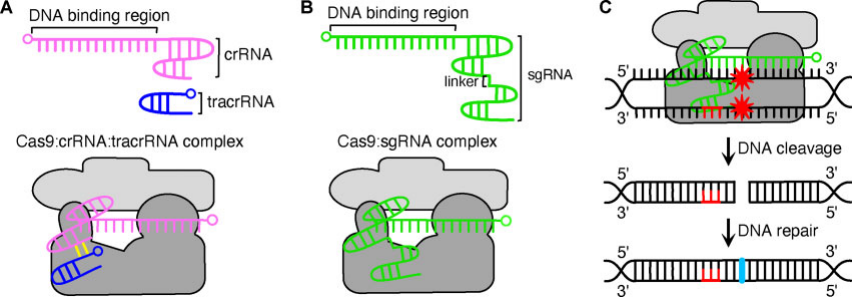
Core components of the CRISPR-Cas9 system. (**A**) In the native system a CRISPR RNA (crRNA; pink) and trans-activating crRNA (tracrRNA; blue), bind together (yellow interactions) to form a gRNA that then complexes with the Cas9 protein (grey). The 5ʹ end of each RNA is denoted by a small circle. (**B**) A sgRNA (green) is produced by fusing a crRNA and tracrRNA using a short linker. This creates a CRISPR-Cas9 system requiring only two components: Cas9 and an sgRNA. (**C**) Function of the CRISPR-Cas9 system. The Cas9:gRNA complex is able to bind DNA and upon recognition of a complementary sequence to the DNA-binding region of the gRNA, double-strand cleavage occurs. Where possible, the cell will attempt to repair this break, which can potentially introduce mutations (cyan bases). Red DNA bases show the PAM and red stars denote DNA cleavage.

Defined originally as an array of DNA repeats in 1987 (1), the exact function of the clustered regularly interspaced short palindromic repeats (CRISPR) remained a mystery until the further discovery of CRISPR-associated (Cas) proteins and RNA elements. This established their combined function as a prokaryotic immune system (2–5), which had evolved to combat invading phages by cleaving and degrading their DNA. The core components are a Cas endonuclease, directed to a DNA target by a multicomponent guide RNA (gRNA) (6, 7), which has since been simplified into a single guide RNA (sgRNA) (8) (**Figure 1**).

The power of the CRISPR system comes from its highly programmable nature that allows it to be easily targeted to virtually any DNA locus by merely placing a complementary sequence within the gRNA. Whilst its built-in functionality has ushered in a new era of genome engineering, CRISPR’s real merit lies in its robustness for significant modification. This has allowed the CRISPR system to be refined as well radically extended to broaden its capabilities. These developments have enabled CRISPR to be used for diverse applications covering gene regulation, large genomic insertions and deletions, accurate base editing, and precise sequence replacement (9–13). This broad and significant utility has resulted in the term ‘CRISPR’ becoming synonymous with CRISPR-Cas systems and their application.

In this review, we explore the development of modified Cas9-based CRISPR systems for genome-editing tasks, and the main approaches used to engineer these functionalities. This includes the mutagenesis of Cas9 domains, redesign of the gRNA, fusion of additional enzymatic domains to Cas9 and the screening of other organisms for naturally occurring CRISPR variants with more desirable features. Our aim is to provide a clear mechanistic overview of how the modular structure of the CRISPR-Cas9 system has facilitated engineering efforts and allowed for a ‘plug-n-play’ type approach to the development of new DNA-targeted functionalities. Whilst the potential benefits of such systems are already starting to be realized, we end by raising caution when considering their deployment and discuss some of the less widely acknowledged scientific, ethical and evolutionary challenges associated with this technology.

It should be noted that other CRISPR systems employing alternative Cas proteins do exist and have begun to gain interest due to their unique and often complementary capabilities. For example, CRISPR-Cas12a-based systems have been shown to simplify multiplexed editing and combinatorial screens due to their ability to process CRISPR arrays directly (14–18). However, Cas9-based systems are by far the most commonly used and modified to date, and so form the focus of this review.

## 2. The native CRISPR-Cas9 system

The CRISPR-Cas9 system is formally classified as a class 2, type II CRISPR system, which was originally derived from *Streptococcus pyogenes* (19). It consists of a Cas nuclease *Sp*Cas9 and a gRNA (8) (**Figure 1**). The gRNA has two components—a trans-activating RNA (tracrRNA) and a CRISPR RNA (crRNA) (6) (**Figure 1A**). crRNA is responsible for recognition and binding of the target DNA region and tracrRNA for crRNA maturation and association with *Sp*Cas9. Alternatively, a chimeric sgRNA which performs both these functions can be used (6) (**Figure 1B**). Once the gRNA binds *Sp*Cas9, *Sp*Cas9 undergoes a conformational change which permits the *Sp*Cas9-crRNA-tracrRNA complex to relocate to the target region and cleave both DNA strands (7). The target region is determined by a 20-nucleotide ‘spacer’ in the crRNA, complementary to the target ‘protospacer’ in the DNA (3, 20). For recognition, the protospacer must be superseded at the 3ʹ end by several nucleotides called the protospacer adjacent motif (PAM). This varies for different Cas proteins; for *Sp*Cas9 it is 5ʹ-NGG-3ʹ (8, 21). Providing there is the correct PAM present at the 3ʹ end of the target locus, engineering a gRNA with a different spacer region allows for targeting of a different genomic location.

When the target region is found, the bases upstream of the PAM are melted and bind to the complementary region of the gRNA (22, 23). Once the complex is bound, the two nucleases produce a double-stranded break (DSB) 3–4 nucleotides (nt) upstream of the PAM (24). The DSB induces the endogenous DNA repair machinery, commonly the non-homologous end-joining pathway (NHEJ). NHEJ is notoriously error-prone, so the break is often fixed incorrectly and the target sequence becomes mutated (25) (**Figure 1C**). Alternatively, the homology-directed repair pathway (HDR) can be used to fix the break using a homologous template to accurately insert a desired sequence (25, 26). HDR is preferred to NHEJ in certain organisms (e.g. *Saccharomyces cerevisiae*) as well as in cells containing a repair template (e.g. cells post S phase of the cell cycle) (27). Recognition of CRISPR’s ability to perform gene knockdown/insertion was the beginning of a series of alterations which would highlight the diverse applications of this system and its derivatives.

Whilst CRISPR can perform efficient cleavage of a target genomic region, a common problem is the presence of non-target cleavage, or off-target effects, particularly in larger genomes (28). The genomic target has 20 nt of complementarity to the spacer region of the gRNA, but mutations of the 5ʹ end of the gRNA still permit efficient cleavage implying only 12–13 nt at the 3ʹ end of the spacer region are critical for specifying the target (21, 24, 25). These essential 13 nt have been dubbed the ‘seed sequence’ (8, 29). Genomic regions with incomplete homology to the spacer region which contain all or most of the seed sequence could be targeted by the Cas9, resulting in off-target effects (30). Detection and prevention of this off-target activity are essential for CRISPR to be used as a therapeutic tool. Efforts utilizing altered, higher-fidelity Cas9 proteins and truncated gRNA (31–33) have been the focus of efforts to reduce such promiscuity and will be discussed later.

To assist with the characterization of CRISPR, large-scale bioinformatic tools have been developed for genomic analysis and specifically the identification of potential editing sites. Complementary biological assays have also been developed to assess off-target cleavage (34). A widely used assay to investigate off-target binding is the T7 endonuclease 1 (T7E1) mismatch detection assay. Despite its widespread use, validations in the literature have exposed the poor accuracy and sensitivity of the T7E1 assay (35). Cleavage by *Sp*Cas9 has been observed at sites with up to five mismatches to the spacer region and even in sites without the 5ʹ-NGG-3ʹ PAM, for example, at those containing 5ʹ-NAG-3ʹ (36, 37).

Computational tools such as Cas-OFFinder and E-CRISP assume that sites with more homology to the spacer region are more likely to be targeted and vice versa, allowing the user to predict potential off-target loci (38, 39). These approaches, however, do not consider off-target sites which do not fit the model’s parameters (40). To alleviate this issue, machine learning methods have recently been shown to offer improved performance (41). Experimentally, Genome-wide, unbiased identification of DSBs enabled by sequencing (GUIDE-seq) provides a robust empirical method for identifying off-target effects and has become widely used (42). A small oligo-nucleotide tag is integrated into DSB sites targeted by NHEJ, and sequencing analysis is used to pinpoint the location of off-target sites. This permits the detection of sites difficult to capture with computational tools due to the complexity of the underlying rules and interactions (38). GUIDE-seq is a simple method to identify sites which have up to six mismatches to the protospacer sequence as well as noncanonical PAMs, giving a broad profile of off-target effects, but is limited by the use of an oligo tag (40, 42). Another example of a genome-wide tool is digested genome sequencing (Digenome-seq) which involves the digestion of genomic DNA with Cas9-gRNA complexes and subsequent deep sequencing to identify identical Cas9 cleavage fragments (43). Analysis is performed on extracted DNA, eliminating the influence of cellular context (e.g. chromatin arrangements, methylation patterns and DNA accessibility). This method is time-consuming as many reads have to be analyzed to identify patterns, and it fails to recognize identical fragments caused by chance (40). Overall, no single method is able to comprehensively analyze off-target effects, therefore, the method employed must be carefully considered on a case-by-case basis. For example, Digenome-seq is appropriate for *in vitro* applications because it is not vulnerable to chromatin arrangements (43), but for *in vivo* applications, GUIDE-seq or the new, multiplexing sister method Tagmentation-based tag integration site sequencing (TTISS) are more sensitive and easier to use (42, 44). For a truly comprehensive understanding of all off-target effects, a multisystem analysis involving both computational and biological approaches is necessary but rarely performed. Whether the field of genome engineering can expect more accurate predictions will largely depend on the ability to combine versatile algorithms with ultrasensitive, genome-wide off-target detection methods and predictive modeling (41, 45).

### 2.1 Naturally occurring variants

**Figure 2.**
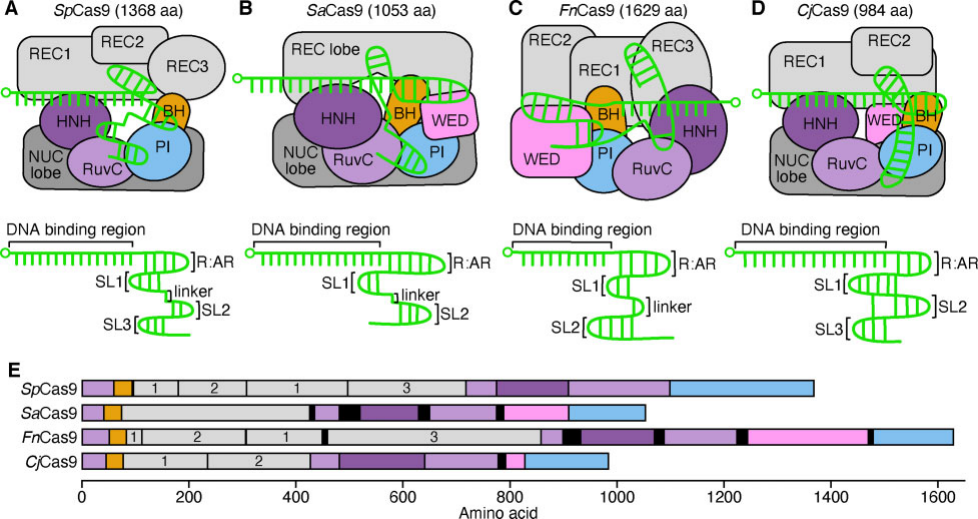
Naturally occurring variants of Cas9 and their respective gRNA structures. Top diagrams show the Cas9:gRNA complex and interactions of the gRNA with core Cas9 domains (labeled). Domains abbreviated as: REC, recognition; NUC, nuclease; BH, bridge helix; PI, PAM-interacting; CTD, C-terminal domain; WED, wedge. HNH and RuvC are nuclease domains. Bottom diagrams show the gRNA structure with the DNA-binding region, major stem loops (SLs) and repeat: anti-repeat (R:AR) duplex highlighted. The 5ʹ end of each gRNA is denoted by a small circle. (**A**) *Streptococcus pyogenes* Cas9 (*Sp*Cas9). (**B**) *Staphylococcus aureus* Cas9 (*Sa*Cas9). (**C**) *Francisella novicida* Cas9 (*Fn*Cas9). (**D**) *Campylobacter jejuni* Cas9 (*Cj*Cas9). (**E**) Domain structure of the Cas9 variants. Linkers are shown by black regions and REC domains are numbered.

CRISPR is a naturally occurring system in prokaryotes, thus different species possess different systems whose variations can be potentially exploited (46). Type I and III systems enlist multiple Cas proteins whereas type II uses a single, Cas9 protein for DNA cleavage (47). Whilst *Sp*Cas9 from *S. pyogenes* is the most heavily studied to date, Cas9 variants from different bacteria with distinct cleavage patterns and PAM requirements are becoming more widely used (**Figure 2**). This includes *Fn*Cas9 from *Francisella novicida* (48), *Sa*Cas9 from *Staphylococcus aureus* (49, 50) and recently the *Campylobacter jejuni* Cas9, the smallest to date (51, 52).

*Sp*Cas9 is a multi-domain protein exhibiting a bilobed structure where the nuclease lobe and the recognition lobe (8, 24) are linked by an arginine-rich bridge helix as well as a disordered linker (8) (**Figure 2A**). The overall shape of *Sp*Cas9 is oblong with two large grooves, to accommodate the DNA:RNA and RNA:RNA complexes. Adaptations of the two previously recognized, adjacent nucleases (HNH (6), named for the three characteristic residues, and RuvC (53)) of the nuclease lobe facilitate much of the diversification of CRISPR’s function (31, 54). Each nuclease cleaves one strand of DNA; RuvC cleaves the non-complementary and HNH the complementary strand (6, 20). Another key component of the nuclease lobe is the C-terminal domain, with a region essential for PAM recognition and binding often called the PAM-interacting (PI) domain (7). Mutagenesis of these domains permits the evolution of CRISPR function.

*Sa*Cas9 has a longer PAM (5ʹ-NNGRRT-3ʹ) than *Sp*Cas9 and is smaller at 1053 amino acids (aa) compared to 1368 aa (49) (**Figure 2B**). Due to its smaller size, *Sa*Cas9 provides valuable information regarding the elements of Cas9 that are essential and those that can be removed or modified without impacting overall function. Characterization of *Sa*Cas9 has shown comparable on-target cleavage to *Sp*Cas9, whilst boasting a higher specificity and easier introduction into cells (55). Both *Sp*Cas9 and *Sa*Cas9 are bilobed, with a nuclease (NUC) and recognition (REC) lobe linked by an arginine bridge and a linker region. They both contain two nuclease domains, HNH and RuvC, and undergo a conformational change when gRNA is bound. However, *Sa*Cas9 only has 17% structural similarity to *Sp*Cas9; key DNA/RNA-binding domains such as the nucleases and the PI domain have been conserved but others such as the REC2 domain are not, suggesting its presence is not crucial for Cas9 function. This demonstrates the flexibility of Cas9’s structure whilst retaining efficacy (55). Despite these differences, it is apparent that *Sa*Cas9 and *Sp*Cas9 share important similarities, and that *Sa*Cas9 is a useful case study for synthetic reduction of *Sp*Cas9 size and complexity, already attempted by the successful removal of the REC2 domain (56).

Another *Sp*Cas9 ortholog is *Fn*Cas9 which produces staggered cleavage and binds less frequently to non-target regions (48, 57) (**Figure 2C**). The non-target strand is cleaved 3–8 bp upstream of the PAM (5ʹ-NGG-3ʹ), whereas the target strand is cleaved 3 bp upstream as by *Sp*Cas9 and *Sa*Cas9, producing overhangs of up to 4 nt and more efficient recruitment of HDR (48). *Fn*Cas9 is considerably larger than *Sp*Cas9 and *Sa*Ca9, comprised of 1629 aa (58). Whilst its larger size may be a hindrance for transfection due to the limited capacity of many delivery systems, *Fn*Cas9’s markedly reduced tolerance of target mismatches makes it a valuable system for precise editing tasks. *Sp*Cas9 tolerates several mismatches of the gRNA in the non-seed region, but just one mismatch at the 5ʹ end of *Fn*Cas9 gRNA is tolerated for successful cleavage (57). This increased specificity means *Fn*Cas9 produces far less off-target cleavage as fewer sites are recognized as ‘target’ (48). *Fn*Cas9 is structurally dissimilar to *Sp*Cas9 and *Sa*Cas9, lacking a bilobed structure and containing distinct REC2 and REC3 domains (**Figure 2C**). REC3 domain mutations have generated high-fidelity Cas9 enzymes (59); these structural differences explain the striking differences in targeting specificity. Despite its increased specificity, it has much lower on-target recognition than *Sp*Cas9 in eukaryotic genomes. As postulated in the literature (57), local chromatin conformations likely affect the access to DNA, a vulnerability not as significant for *Sp*Cas9. To eliminate this problem *Fn*Cas9 has been used alongside a catalytically dead *Sp*Cas9 (*Sp*dCas9) to enable access and subsequent DNA cleavage (57). Such problems are not present when used in prokaryotes where *Fn*Cas9 has been shown to function effectively (60).

Finally, *Cj*Cas9 is the smallest ortholog characterized to date at only 984 aa, making it suitable for size-restricted delivery methods such as those using adeno-associated viruses (AAV) (**Figure 2D**). It has a bilobed structure, akin to *Sa*Cas9 and *Sp*Cas9, with a simplified REC lobe and size-reduced NUC lobe (52) (**Figure 2D**). Initial studies showed recognition of a 5ʹ-NNNNACA-3ʹ PAM (46) or the more promiscuous 5ʹ-NNNVRYM-3ʹ (52) providing an assortment of target sites. However, recent studies have found a requirement for an 8th cytosine at the 3ʹ end, suggesting 5ʹ-NNNNRYAC-3ʹ (51) and 5ʹ-NNNNACAC-3ʹ sequences (61). Tested against *Sa*Cas9 in human cells, *Cj*Cas9 was found to be more specific with comparable efficiencies to some other variants, excluding *Fn*Cas9 (51). However, due to discrepancies in the PAM recognition sequences and limited research into the structure and mechanism of *Cj*Cas9, care should be taken when placing confidence in this finding.

Comparisons of each Cas9 ortholog and their respective sgRNA have also revealed several structural and functional differences (**Figure 2**). The essential region of the sgRNA consists of a DNA-binding region, the repeat: anti-repeat duplex (R:AR) and at least two stem loops. Removal of stem loop 1, which has extensive interactions with Cas9, prevents cleavage, so its presence is essential (6, 49). In contrast, removal of loops 2 or 3 decreases efficiency, without abolishing cleavage (24). Stem loop 2 interacts with the PI and RuvC domains in *Sa*Cas9 and *Sp*Cas9, and the REC domains in *Fn*Cas9 and *Cj*Cas9 (7, 49, 52, 56, 58). *Sa*Cas9 and *Sp*Cas9’s sgRNAs exhibit the greatest similarity, particularly regarding cognate Cas9 interactions with the lack of stem loop 3 in *Sa*Cas9 the defining key difference (49). This further highlights the minimalism of *Sa*Cas9 compared to *Sp*Cas9 because of the reduction of non-essential elements like stem loop 3 and the REC2 domain (55). *Fn*Cas9 and *Cj*Cas9’s sgRNAs are structurally distinct to *Sa*Cas9 and *Sp*Cas9, with the same core region but some unique features. For instance, *Fn*Cas9 has a longer, U shaped linker, contrasting with the shorter, single-stranded linker present in *Sa*Cas9 and *Sp*Cas9 (58). The novel structural arrangement of *Cj*Cas9’s gRNA forms a triple helix between stem loops 1, 2 and 3 (52). The relevance of this structure is still unknown due to a lack of comprehensive structural studies of *Cj*Cas9 complexes.

The domains of each Cas9 distinctly interact with their associated sgRNAs due to the slight differences in sgRNA structure (49) (**Figure 2**). The stark differences between *Sp*Cas9 and its orthologs demonstrate the diversity of naturally occurring Cas9 systems and their varying characteristics. Whilst the four orthologs discussed here have been characterized and established as potential genome-editing tools, their testing still pales in comparison to *Sp*Cas9 and we expect that further characterization experiments will be needed before their deployment. Even so, the differences in mechanism and function seen across these variants clearly highlight the wealth of preexisting systems available that may be suitable for many applications.

## 3. Modifying CRISPR-Cas9 to enhance performance

### 3.1 Modification of gRNAs

The CRISPR-Cas9 system requires a tracrRNA and a crRNA for target complementarity and complex maturation. To simplify use, a single chimeric guide RNA (sgRNA) is generally used to describe the dual-tracrRNA:crRNA structure (**Figure 2**, bottom row). As established by Jinek *et al.* (6), a seed region (13 nt of complementarity between the crRNA and the 3ʹ end of the protospacer sequence) and a GG dinucleotide at the 3ʹ end of the PAM are essential for sequence-specific recognition and cleavage. By fusing the 3ʹ end of the crRNA to the 5ʹ end of tracrRNA this study simulated the tracrRNA:crRNA duplex formed in nature, inducing a Cas9 open conformation and directed DNA targeting. In this study, the chimeric gRNA produced cleaved all five expected targets *in vitro* and has since been widely used, confirming its efficacy (6). Such mimicking of nature’s gRNA design is a great example of how simple biotechnological approaches can yield more streamlined genetic engineering systems.

Another modification involves truncating the gRNA such that it contains <20 nt of complementarity to a target locus. Truncated gRNAs or tru-gRNAs have demonstrated significantly lower off-target activity compared to full-length sgRNAs due to a reduction in binding affinity and greater mismatch intolerance (39, 62). As demonstrated in two human cell lines, the specificity of tru-gRNAs as compared to wild-type was estimated to be >5000-fold higher (33). Such estimates are supported by the finding that additional nucleotides added at the 5ʹ end of gRNA increase binding affinity for off-target sites (28). Using the same study systems, it has been shown that positive synergism between tru-gRNAs and paired Cas9 nickases permits a further reduction in off-target activity, demonstrating the promise of the additive effects when combining modifications. Beyond sequence changes to gRNAs, another method that has been used to improve editing efficiency is the chemical modification of key nucleotides. Chemically synthesized and modified sgRNAs have shown significantly improved editing efficiencies in human primary T cells and CD34+ hematopoietic stem and progenitor cells (63). The ability for Cas9 to handle significant modifications has enabled the effective use of gRNAs with >80% ribose substitutions and at least one chemical modification (e.g. 2ʹ-O-methyl, 2ʹ-Fluoro, phosphorothioate) at every nucleotide position (63). Such modifications are useful as they can help ensure metabolic stability and reduce the chance of nanoparticle formation, which can elicit an immune response. Furthermore, such modifications offer the ability to use chemical conjugates as a means to target the cell-surface and improve uptake (64).

### 3.2 Modification of Cas9

**Figure 3.**
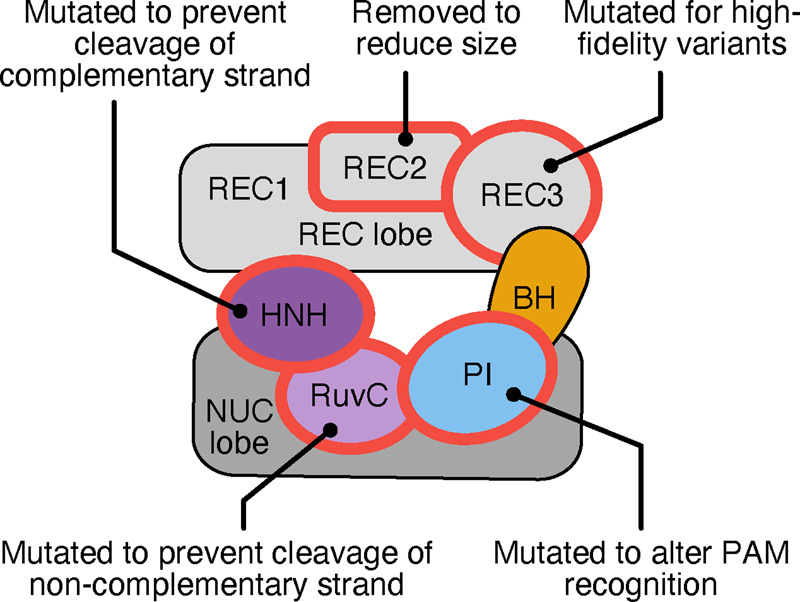
Key domains of Cas9 and the effect of modifications of each on phenotype. Domains abbreviated as: REC, recognition; NUC, nuclease; BH, bridge helix; PI, PAM-interacting. HNH and RuvC are nuclease domains. Thick red outlines indicate domains which have been modified.

**Figure 4.**
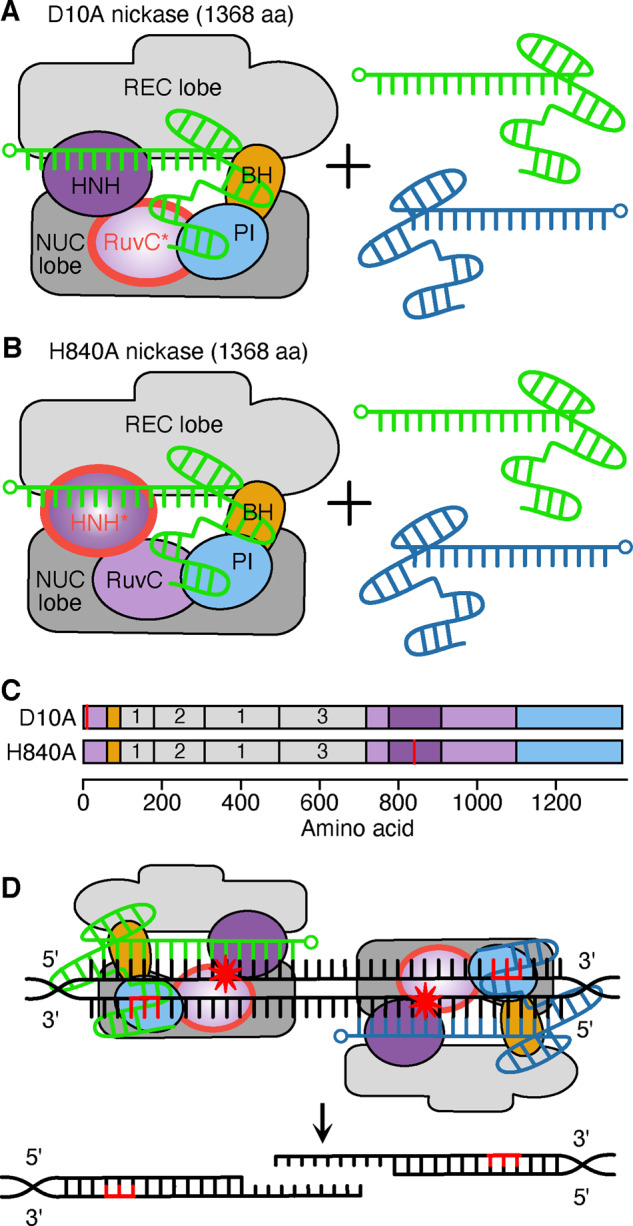
Cas9D10A and Cas9H840A nickase systems. (**A**) The Cas9D10A nickase system which nicks the complementary strand. This Cas9D10A is used in conjunction with a pair of guides to target each strand independently. The 5ʹ end of each gRNA is denoted by a small circle and inactive domains are outlined in red. Domains abbreviated as: REC, recognition; NUC, nuclease; BH, bridge helix; PI, PAM-interacting. HNH and RuvC are nuclease domains. (**B**) A complementary Cas9H840A nickase system is able to nick the non-target strand. Again, this system is normally used with two complementary guides to target each strand of DNA. (**C**) Domain structure of the nickase system. Mutations are shown by red lines and the three REC domains are numbered. (**D**) Example of the Cas9D10A nickase system targeting two regions to create complementary nicks on opposite strands. The PAM is shown in red and red stars denote DNA cleavage.

Another method to improve performance is through modification of the Cas9 enzyme itself (**Figure 3**). Analysis of CRISPR-Cas9 variants and their resultant cleavage products established RuvC and HNH nuclease-mediated cleavage of the non-complementary and complementary strand, respectively (6, 20). As double-stranded cleavage often favors the inaccurate NHEJ pathway (depending on the organism, cell type and stage in the cell cycle), single-stranded cleavage (or ‘nicking’) is favorable for efficient targeted replacement (27). A deactivating mutation in the catalytic residues of one of the nucleases causes the Cas9 to cleave only one strand of the target DNA. Such nicking permits accurate HDR or base excision repair (BER) (65, 66). Two nicking variants (henceforth nickases) were engineered by an aspartate to alanine substitution in the active site of the RuvC domain to produce Cas9D10A and histidine to alanine substitution in the HNH domain to produce Cas9H840A (20, 25, 31). The benefits of these are twofold: they produce precise nicks in the DNA and exhibit decreased affinity to off-target loci (31). When a DSB is required, a nickase can be used with two different gRNAs that target each strand of the DNA. When both nicks are performed a staggered cleavage site is produced (**Figure 4**) (67). This dual nicking strategy has been shown to have comparable on-target cleavage to *SpCas9* whilst discriminating off-target sites more effectively, however, requires the presence of two neighboring PAM sites which limits the number of potential editing sites (68). Continued editing of nickases forms the basis of many other CRISPR editing systems that will be explored in the next section. Additional reductions in off-target effects have also been achieved by controlling the expression and stability of the Cas9 protein. For example, increasing the degradation rate of Cas9 by adding an ubiquitin-targeting signal added to the N-terminus has been shown to decease mosaicism in monkey embryos (69). Furthermore, the addition of an N-terminus geminin tag to Cas9 has been used to regulate Cas9 concentration in response to the cell cycle allowing the editing capacity to be maintained while greatly reducing neurotoxicity (70).

As a mutation in one of the nuclease domains can alter Cas9 from a dsDNA endonuclease to an ssDNA nickase, mutation of both domains will remove all cleavage activity. An *Sp*Cas9 enzyme containing the H840A and D10A mutations is catalytically dead (dCas9) (6, 71), but is still able to target and bind DNA. dCas9 has been shown to be a versatile tool and can be tethered to other molecules such as other enzymes (9) or used to visualize target affinity without cleavage (54). Such an approach has enabled the development of a programmable DNA methylation system formed from a dCas9 protein fused to a DNA (cytosine-5)-methyltransferase 3A. This particular system permitted up to 50% methylation for targeted CpG dinucleotides in HEK293T cells (72) and a better understanding of the influence chromatin organization and dynamics plays has on gene expression. Particularly in human cells, programmable DNA methylation systems allow for the visualization of specific genetic loci via a dCas-eGFP fusion and fluorescence microscopy (73).

Furthermore, dCas9 has become widely used in regulating gene expression through CRISPR interference and activation (CRISPRi and CRISPRa, respectively) (74, 75). Interference of gene expression is generally achieved by targeting the dCas9 protein to promoter regions to sterically block the initiation of RNA polymerase (76). Additional, repression domains (e.g. KRAB) can also be fused to the dCas9 to enhance repression (77). This ability to inhibit but not completely turn off gene expression has made CRISPRi a valuable tool for knock-down screens where Cas9 is not suitable (e.g. due to genotoxicity) (78). Activation of gene expression has been similarly achieved by fusing transcription activating domains (e.g. VP64 for human cells or SoxS for *Escherichia coli*) to dCas9 (76, 79), or by modifying the sgRNA and using an RNA-binding protein (e.g. MS2 coat protein) fused to an activator domain that can then be targeted to this sgRNA (80). In both cases, targeting these systems to regions upstream of a promoter without blocking transcription initiation enables activation of the downstream gene.

**Figure 5.**
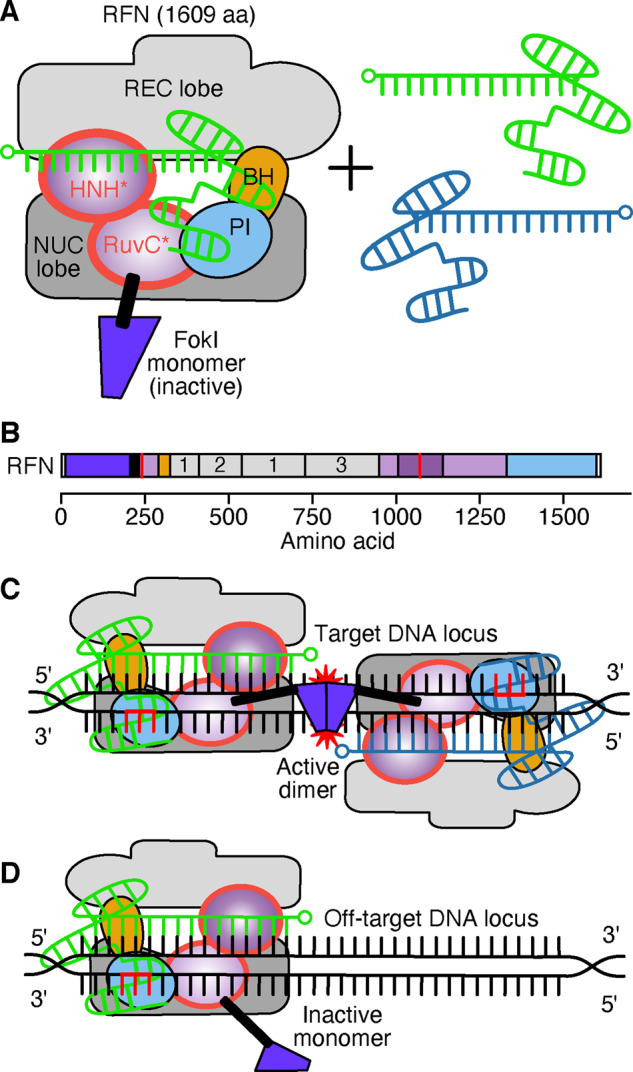
An RNA-guided FokI Nuclease (RFN) system. (**A**) An RFN system consists of a dCas9-FokI fusion and two gRNA (green and blue) with targets ∼15 bp apart. Two FokI monomers (blue) are required for the active dimer (purple) to cleave DNA, so off-target binding of a single RFN does not (usually) result in cleavage. Domains abbreviated as: REC, recognition; NUC, nuclease; BH, bridge helix; PI, PAM-interacting. HNH and RuvC are nuclease domains. Domains outlined in red are inactive. The 5ʹ end of each gRNA is denoted by a small circle. The PAM is shown in red. Linkers are denoted by white rectangles. (**B**) Domain structure of the RFN. Linkers and nuclear localization signals (NLSs) are denoted by black and white regions, respectively, and mutations are shown by red lines. The three REC domains are numbered. (**C**) Two RFNs bound in an active conformation to a target DNA locus. Red stars denote DNA cleavage. (**D**) Single inactive RFN bound to an off-target DNA locus.

An additional application of dCas9 concerns fusion to a FokI nuclease, an endonuclease which is strictly dependent on dimerization for cleavage activity (81). This fusion enlists a long, flexible linker with between 5 and 25 residues (e.g. GGGGS)_5_ fusing the FokI endonuclease to the Cas9 N-terminus (81–83). The RNA-guided FokI Nuclease (RFN) system consists of a dCas9-FokI fusion and two different gRNAs (84). These gRNAs must have specificity to the target region, and both must be bound to their respective loci to allow for a functional FokI dimer to form and cleavage to take place. When there is off-target binding by one gRNA:Cas9 complex, the FokI monomer remains inactive and cleavage does not occur (81) (**Figure 5**). The use of these alternative, exogenous nucleases creates a highly specific system with significantly lower indel frequencies when compared to wild-type Cas9 nucleases and the use of single gRNAs (83). However, RFNs are limited for genome-wide application due to the required presence of PAM sequences either side of the protospacer regions (5ʹ-CCNN_20_-3ʹ and 5ʹ-N_20_NGG-3ʹ) as well as 14–17 bp between these (82). This fusion system is also very large, limiting its application in AAV delivery methods (85). Efforts have been made to use the smaller *Sa*Cas9-based system instead of *Sp*Cas9, reducing the size and simplifying delivery (82). Despite some documented successes (86, 87), it is worth noting the range of confounding effects associated with the different delivery methods. For example, a complication when employing lentivirus vectors concerns long-term Cas9 expression which promotes the likelihood of off-target effects (88). In contrast, Cas9 ribonucleoproteins are limited by transient expression and possible reduced on-target activity (89).

### 3.3 Mutation of REC3 domain

Targeted mutagenesis of other Cas9 domains has also been performed to find additional useful modifications. For example, as DNA binds between the HNH and REC domains, mutations of the positively charged residues of REC3 to alanine could reduce binding affinity making the Cas9 more strongly discriminate between target and off-target regions (90). Using this knowledge, a high-fidelity Cas9, *Sp*Cas9-HF1 was produced via mutation of four DNA-interacting REC3 residues to alanine (N497A/R661A/Q695A/Q926A), with comparable on-target cleavage to *Sp*Cas9 (32). Despite the reduction in off-target mutations as quantified by GUIDE-seq, this variant was incompatible with the optimized truncated gRNA demonstrating a case where independent enhancements could not be combined.

A failure to completely abolish off-target activity in *Sp*Cas9-HF1 led to further screening of REC3 mutants *in vivo* and the development of another highly specific *Sp*Cas9 variant, dubbed ‘evoCas9’ (59). This variant outperforms *Sp*Cas9-HF1 in distinguishing between on and off-target sites and has better compatibility with optimized gRNAs.

### 3.4 Directed evolution for altered PAM specificity

Alterations to the nuclease and recognition domains have been shown to improve target specificity and efficiency. However, *Sp*Cas9 is still limited to targeting of genomic regions containing the 5ʹ-NGG-3ʹ PAM (6), whose number may be further reduced by local chromatin or methylation patterns preventing Cas9 access to the site (25). PAM specificity is conferred by several residues of the PI domain, specifically *Sp*Cas9 arginine residues 1333 and 1335 which interact with the two guanine nucleotides of the PAM (7). Motivated by this, several studies have focused on mutagenizing this domain to change the PAM recognized by Cas9. An attempt in 2014 substituted the two critical guanine-recognizing residues which interact with adenine from arginine to glutamine in an attempt to modify *SpCas9* recognition to a 5ʹ-NAA-3ʹ PAM (91). This effort was unsuccessful and the R1333Q/R1335Q variant produced failed to cleave DNA *in vitro*. It was concluded that additional mutations were likely required for successful alteration of PAM recognition.

Building on this work, Kleinstiver *et al.* employed a positive selection approach where survival of bacteria was only guaranteed by Cas9 cleavage of a toxic gene (50). This produced two main variants: VQR (D1135V/R1335Q/T1337R) which recognized 5ʹ-NGAN-3ʹ and 5ʹ-NGCG-3ʹ PAMs and VRER (D1135V/G1218R/R1335E/T1337R) which recognized the 5ʹ-NGCG-3ʹ PAM. The T1337R mutation was found to be a gain of function, contrasting with the loss-of-function mutations utilized by other domain mutagenesis studies. This specific gain of function permitted Cas9 recognition of a fourth PAM base which increased the stringency of binding and reduced off-target effects compared to wild-type *Sp*Cas9 (50). These evolved *Sp*Cas9 variants with altered PAM specificities are still limited to one or two PAMs.

To expand PAM recognition, focus has shifted to generating *Sp*Cas9 variants able to target multiple PAMs. So far, positive selection has been used to find useful mutagenized *Sp*Cas9 variants using phage assisted continuous evolution (21). Such variants, dubbed ‘xCas9’ nucleases, had a different pattern of mutations than the rationally developed variants which covered the entire *cas9* gene (7, 50). xCas9-3.7 showed the best cleavage efficiency, with a high indel formation of DNA adjacent to 5ʹ-NG-3ʹ, 5ʹ-GAA-3ʹ and 5ʹ-GAT-3ʹ PAMs as well as comparable activity to 5ʹ-NGG-3ʹ with *Sp*Cas9 (21). Together with the broader on-target specificity, xCas9-3.7 produced less off-target cleavage than *Sp*Cas9, demonstrating the potential merits of using an engineered Cas9 rather than the native system.

Mutation of the PI domain in this way is not limited to *Sp*Cas9 and has been performed in *Sa*Cas9 to similar effect. Using an analogous bacterial selection approach, mutated *Sa*Cas9 variants were tested for their efficiency for 5ʹ-NNNRRT-3ʹ PAM loci cleavage. Results showed that an E782K/N968K/R1015H variant called *Sa*KKH was functional and that this variant disrupted 5ʹ-NNGRRT-3ʹ sites (and off-target loci) at a similar efficiency to wild-type *Sa*Cas9 whilst also cleaving sites adjacent to 5ʹ-NNARRT-3ʹ, 5ʹ-NNTRRT-3ʹ and 5ʹ-NNCRRT-3ʹ (92).

## 4. Plug-n-play CRISPR-Cas9 modules

### 4.1 Base editing

NHEJ-based methods are useful for the downregulation or knock-out of genes, but for more precise editing the less error-prone HDR is preferred. HDR has been shown to work alongside the CRISPR system and in theory can induce a range of genome edits, but is hard to employ *in vivo* due to the difficulties associated with successful delivery of both the editing machinery and template DNA (27). Additionally, both of these DNA repair pathways rely on the generation of DSBs, which can result in inadvertent genomic alterations, pathogenic lesions and deleterious tumor suppressor p53 activation responses (93). Single-stranded nicks are repaired by the high-fidelity BER pathway, making this cleavage pattern preferable for specific base changes (66).

**Figure 6.**
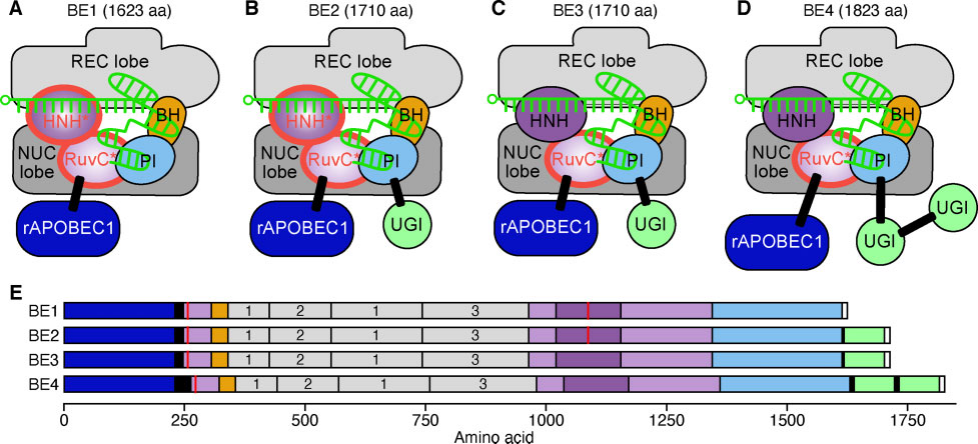
Base editing systems. (**A**) Base editor 1 (BE1) consists of a *Sp*dCas9 with a cytidine deaminase (rAPOBEC1) fused to its N-terminus. Domains abbreviated as: REC, recognition; NUC, nuclease; BH, bridge helix; PI, PAM-interacting. HNH and RuvC are nuclease domains. (**B**) Base editor 2 (BE2) is similar to BE1 but includes an additional uracil glycosylase inhibitor (UGI) fused to the C-terminus. (**C**) Base editor 3 (BE3) is similar to BE2 but includes the catalytic activity of the HNH nuclease domain restored, to allow target strand nicking. (**D**) Base editor 4 is as BE3 but with longer linker proteins and an additional UGI fused to the C-terminus. The 5ʹ end of each gRNA is denoted by a small circle. Linkers are denoted by white rectangles. Mutated domains are outlined in red. (**E**) Domain structure of the base editors. Linkers and nuclear localization signals (NLSs) are denoted by black and white regions, respectively, and mutations are shown by red lines. The three REC domains are numbered.

Studies of the mechanism of Cas9 cleavage have revealed that the displaced DNA strand is unbound, this finding coupled with the need to more accurately alter genetic sequences led to the development of base editors (94) (**Figure 6**). A simple CRISPR base editor consists of a dCas9 protein, a sgRNA and a base-editing enzyme (e.g. cytidine deaminase) (95). Cytidine deaminases catalyze the conversion of cytosine to uracil (96) and the rat cytidine deaminase (rAPOBEC1) has been used in several systems due to its high activity. To localize rAPOBEC1 to a target site in DNA and create the first base editor (BE1), rAPOBEC1 was fused to dCas9 via an XTEN linker which is commonly used in FokI-dCas9 fusions (83, 97) (**Figure 6A**). BE1 is able to deaminate 5 bases at the 5ʹ end of the protospacer and was found to have a 50–80% efficiency *in vitro*, but only 0.8–7.7% in human cells (71). This discrepancy was attributed to the endogenous DNA repair machinery, specifically uracil DNA glycosylase (UDG), which reverses the UG pair to a CG pair (71). To combat this, a uracil DNA glycosylase inhibitor (UGI) was attached to the C-terminus of BE1, to create the second base editor variant BE2 (**Figure 6B**). This alteration increased editing efficiencies in human cells 3-fold as UDG activity was drastically reduced (71). Both these editors are only active on the strand containing the cytosine so to broaden the editors’ function dCas9 was modified to create variant BE3 that acted as a nickase targeting the non-edited strand (**Figure 6C**). BE3 was 2- to 6-fold more efficient in creating cytosine to thymine transitions than BE2. All three editors showed off-target binding, but no base editing was found to have occurred at these sites and indel formation was significantly less than that induced by Cas9-mediated DSBs. A further development produced an additional base editor variant BE4 which included three alterations to BE3 (**Figure 6D**). The linkers fusing the rAPOBEC1 and UGI proteins to Cas9 were extended to 32 and 9 aa, respectively, and an additional UGI was added to the C-terminus with a 9 aa linker (98). BE4 showed higher C to T editing efficiency and product yield compared to BE3. The evolution of this base editor system highlights the robust nature of the Cas9 protein to the ‘plug-n-play’ for additional functional modules in a rational way.

Another study which used this combined approach employed a *Sa*Cas9 nickase instead of *Sp*Cas9 in a BE3 variant, *Sa*BE3 (99). As previously described, *Sa*Cas9 is much smaller than *Sp*Cas9 (49) and recognizes a 5ʹ-NNGRRT-3ʹ PAM. The creation of a base-editing system with this different nickase allowed for targeting of not only 5ʹ-NGG-3ʹ but also 5ʹ-NNGRRT-3ʹ PAMs, increasing the number of potential editing sites. *Sa*BE3 also possesses other benefits, such as an increased editing efficiency on target as well as base editing outside of the expected activity window compared to the *Sp*Cas9-based BE3 (71, 99). Furthermore, Kim and colleagues utilized *Sp*Cas9 variants with altered PAM specificities, specifically VQR and VRER (described previously) and EQR from the same study (50), as well as an engineered *Sa*Cas9 variant, *Sa*KKH (92). All these variants had editing efficiencies of up to 50% for sites with relevant PAMs, with *Sa*KKH-BE3 editing up to 62% of target sites. *Sa*BE3 and *Sa*KKH-BE3 had a similar off-target activity to *Sp*Cas9 whereas EQR-BE3 and VQR-BE3 showed markedly reduced levels (99). These data again highlight the merits of combining CRISPR-Cas9 modifications to extend functionalities.

### 4.2 Prime editing

**Figure 7.**
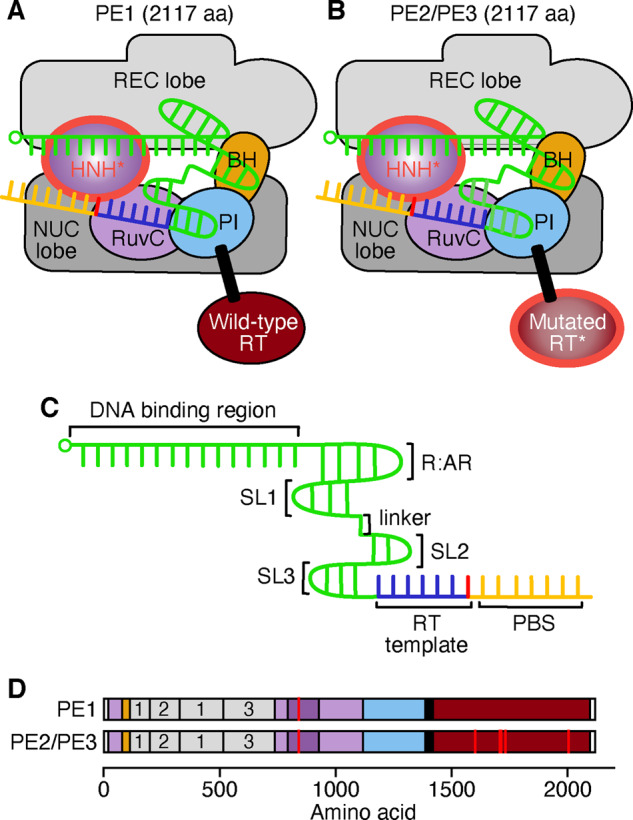
Prime editing systems and pegRNA. (**A**) Prime editor 1 (PE1) consists of an H840A nickase with a flexible linker fusing an M-MLV wild-type (WT) reverse transcriptase (RT; red) to the C-terminus. Domains abbreviated as: REC, recognition; NUC, nuclease; BH, bridge helix; PI, PAM-interacting. HNH and RuvC are nuclease domains. Linkers are denoted by black rectangles. Mutated domains are outlined in red. (**B**) Prime editor 2 (PE2) is similar to PE1 but contains a mutated/engineered RT rather than the WT variant. Prime editor 3 is identical to PE2 but makes use of an additional gRNA targeting the unedited strand, allowing for increased editing efficiency. This second gRNA for PE3 is not a pegRNA and does not contain any modification. (**C**) The pegRNA consists of a seed region and sgRNA (green) with a primer binding site (PBS; dark yellow) and repair template (RT template, blue) containing a base edit (red). Major stem loops (SLs), linker and repeat: anti-repeat (R:AR) duplex are also labeled. The PBS binds to the nicked strand for initiation of repair via RT, using the repair template. The 5ʹ end of each gRNA is denoted by a small circle. (**D**) Domain structure of the prime editors. Linkers and nuclear localization signals (NLSs) are denoted by black and white regions, respectively, and mutations are shown by red lines. The three REC domains are numbered.

A similar combinatorial approach was used to create another form of more complex editing machinery. So-called, prime editing combines the functionalities of a Cas9 nickase, reverse transcriptase (RT) and unique prime editing gRNA (pegRNA) (**Figure 7**). By combining these elements more precise changes to DNA can be made that go beyond the capabilities of other base editors (e.g. transversion point mutations, insertions, deletions) (11). The pegRNA is novel, as it both guides the Cas9-gRNA complex to the target and provides the sequence substrate for the RT to rewrite into the genome. The first prime editor PE1 consisted of a wild-type M-MLV RT attached to the C-terminus of H840A nickase (**Figure 7A**). PE1 was able to generate transversion mutations at efficiencies of up to 5.5% and insertions and deletions of up to 17% (11). To increase the efficiency of PE1, a second prime editor variant PE2 was produced by incorporating five RT mutations designed to enhance binding affinity (**Figure 7B**). PE2 had increased efficiency of insertions and deletions and up to 5.1-fold increases in efficiency of targeted point mutations as compared to PE1. The further prime editor PE3 used the PE2 protein machinery alongside an additional sgRNA targeting the non-edited strand (**Figure 7C**). This simple modification increased editing efficiency by 1.5- to 4.2-fold, which is thought to be due to the edited strand acting as a template for non-edited strand repair (11).

## 5. Challenges

### 5.1 Inconsistent off-target detection methods

Precise detection of off-target activity is crucial if CRISPR technology is to be used more widely and especially in a clinical setting (100). However, many existing methods have differing sensitivities (101) making comparisons between studies difficult (e.g. CIRCLE-seq has been shown to identify more off-target cleavage sites compared to GUIDE-seq and Digenome-seq, whilst Sanger sequencing identifies more compared to T7E1 assays). Furthermore, many of the original CRISPR-Cas9 results that the field has been built upon utilized suboptimal detection methods (102, 103). A further complication concerns the disagreements between *in vitro* and *in vivo* results, which have been reported even for some of the most robust methods developed (65). Together these problems make comparisons and decisions on use difficult. Therefore, moving forward it will be essential that more reliable off-target detection methods are developed, as well as revisiting historic results to verify their accuracy.

### 5.2 Limitations in CRISPR research

Another factor hampering our understanding and comparison of CRISPR-Cas9 systems is the lack of standardized studies and benchmarking (104). Most studies to date have made use of different genetic targets of a limited number, with experiments performed under a variety of environments (i.e. *in vivo/in vitro*) and conditions. While this is understandable given the often-applied focus of research to a particular disease, it does, however, make clear comparisons between methods impossible and further hinders effective reuse of data. In other areas like sequencing, standardized materials have been developed to allow for the robust benchmarking of methods (e.g. synthetic RNA libraries to assess the accuracy of read counts (105) and defined microbial communities to test metagenomic inference from mixed pools of organisms (106)). Although difficult given the broad potential applications of CRISPR, having a set of standardized organisms, cell lines, targets and conditions that cover a wide variety of possibilities would greatly aid in the unbiased assessment of new methods and ensure results can be directly compared. It should be noted that such issues with standardization do not only affect CRISPR research but are a challenge across the whole of the synthetic biology and bioengineering fields.

An additional bias when assessing CRISPR use is the relatively young age of the technology. Most studies to date have focused on demonstrating successful proofs-of-concept with little concern for the longer-term implications. Furthermore, those moderately longer-term studies that do exist have largely focused on ill-effects, e.g., effects on the tumor suppressor gene, *p53* (107, 108). Clearly, this handful of examples does not paint a full picture and the reality is that we have a very limited and biased understanding as to the long-term consequences of CRISPR use (109). Ensuring we are aware of these biases will be crucial when considering possible future deployment into the clinic or the wider environment (e.g. through gene drives (110, 111)).

### 5.3 Ethical, societal and evolutionary concerns

Parallel to scientific advances, ethical and societal concerns have also grown around preclinical research, somatic cell editing, and germline alterations using CRISPR-Cas9. The main focus of these surround germline editing; the work of He Jianku in 2018 that led to the CRISPR-baby scandal re-emphasized the dangers of not regulating this technology (112). In Jianku’s work, the *CCR5* gene was largely disabled to confer protection from HIV infection. However, the pleiotropic role of *CCR5* suggests likely undesirable long-term side effects (113). Understanding the full impact of any germline edit is incredibly difficult. It dictates the fate of individuals, forbids consent of future offspring and potentially exposes the lineage to off-target mutagenesis risks (114, 115), making it ethically questionable in most cases. For those cases where it might be acceptable, open and balanced discussions at a societal level must be performed to ensure this technology is used in an understood and agreed manner. Such ethical considerations should also extend to that of the manufacturing sectors (e.g. agriculture, pharmaceutical and chemical). Although there is promise for CRISPR technologies here, genetically modified food controversies, arguments concerning human health and environmental implications threaten such uses.

From a Darwinian perspective, CRISPR technologies are a powerful means by which individuals could eradicate genes they deem as deleterious from a population. Furthermore, the decision to remove one deleterious gene will likely make it easier to justify the removal of another (116). This ‘slippery slope’ ultimately leads to removal of genes in a biased manner, moving from a situation where genome editing is used for medical necessity to one with a selfish purpose, such as enhancing one’s offspring (117). The ability to select for and against traits would allow humans to act as mediators of natural selection, and bioethicists fear that such control tempts a backlash from nature (118). What form this might take has yet to be fully understood but has drawn recent attention (113, 119). Longer-term, the ability to delete variation and distort heritability, two factors influential of selection, may eventually call for a revised theory of natural selection with ethical and societal implications that go far beyond clinical applications.

## 6. Conclusion

In this review, we have shown how robust the CRISPR-Cas9 system is to modifications and extension, allowing its functionality to be tailored for a broad array of genome-editing tasks in virtually any organism (**Table 1**). The rapid development of these systems was made possible by the highly modular structure of both the Cas9 protein and its associated gRNA that allowed in many cases for directed mutations to have a desired impact on the systems overall function. This bodes well for the engineering of other non-Cas9-based CRISPR systems that may better suited to other tasks such as multiplexed DNA editing (e.g. Cas12a (14, 18)) or the localization of enzymatic activities to RNAs (e.g. Cas13 (161)).

**Table 1.** Organisms and key cell types targeted using CRISPR-Cas9 systems

System	Target	Notes	Refs.
*Fn*Cas9	Mouse (kidney cells^a^) Human (PBMCs,^a^ kidney cells,^a^ liver carcinoma cells^a^)	Comparable indel formation to *Sp*Cas9 with little to no off-target activity Hirano *et al.* found it only worked when provided as an RNP complex	(48, 58, 120)
*Sp*Cas9	Human (embryonic kidney cells^a^) Mouse (embryonic stem cells) Rat (one-cell stage embryos) Zebrafish (one-cell stage embryos) *Drosophila melanogaster* (embryonic cells) *Arabidopsis thaliana* (one-cell stage embryos) Liverwort (gametophytes) *Caenorhabditis elegans* (germline syncytia) Yeast (*Saccharomyces cerevisiae, Pichia pastoris*, …) Gram-postitve/-negative bacteria (*Escherichia coli, Streptomyces lividans, Streptococcus pneumoniae, Bacillus subtilis*, …)	First CRISPR-Cas9 system to be used *in vivo* Has been used to edit genomes of a broad variety of organisms across most kingdoms of life	(121–133)
*Cj*Cas9	Mouse (retinal cells, muscle cells, pancreatic cells)	Comparable indel formation to *Sp*Cas9 and no off-target activity No signs of toxicity 14 months after editing	(51, 134–136)
*Sa*Cas9	Mouse (hepatic cells, embryo fibroblasts) Human (embryonic kidney cells^a^) *Arabidopsis thaliana*	No observable off-target activity at candidate sites (mouse and human) No signs of toxicity 1-month post manipulation (mouse and human) *Sa*Cas9 gave more DSB induction than *Sp*Cas9 in *Arabidopsis*	(137–139)
*Sp*Cas9 Nickase	Human (HeLa cells^a^) Brown Norway rat (midbrain neurons) *Arabidopsis thaliana*	Can be used in a ‘paired nickase’ approach for increased targeting specificity Used in many more studies in more complex systems e.g. base editors	(68, 140, 141)
*Sp*Cas9 RFN	Human (osteosarcoma cells,^a^ embryonic kidney cells^a^)	Less off-target cleavage than wild-type (WT) *Sp*Cas9 Has limited target sites due to extra requirements Greater specificity than the paired nickase approach	(81, 83, 142)
*Sa*Cas9 RFN	Human (embryonic kidney-GFP cells,^a^ embryonic stem cells^a^)	More restrictive requirements than *Sp*Cas9 RFNs, but different PAM required so different target sites available Can be paired with a *Sp*Cas9 RFN monomer for a heterodimer, higher efficiency than *Sa*Cas9 RFN dimer	(81)
*Sp*Cas9-HF1	Human (osteosarcoma cells^a^ embryonic kidney cells^a^) Potato (protoplasts) Chicken (embryo fibroblasts)	70% of WT *Sp*Cas9’s target sites were targeted by *Sp*Cas9-HF1 No activity at the off-target sites where WT *Sp*Cas9 was active	(32, 143–145)
evoCas9	*Saccharomyces cerevisiae* Human (embryonic kidney cells^a^)	Higher targeting efficiency than WT *Sp*Cas9 Significantly more on-target cleavage than *Sp*Cas9-HF1 Both *Sp*Cas9-HF1 and evoCas9 had almost no off-target cleavage, evoCas9 slightly less	(59, 145)
VQR/VRER *Sp*Cas9	Zebrafish (one-cell stage embryos) Human (osteosarcoma cells^a^) *Escherichia coli* *Caenorhabditis elegans* Rice *Arabidopsis thaliana*	VQR targets 5ʹ-NGAN-3ʹ and 5ʹ-NGCG-3ʹ PAMs, VRER 5ʹ-NGCG-3ʹ Both variants could target sites which WT *Sp*Cas9 cannot VRER showed increased fidelity to WT *Sp*Cas9, possibly because of the 4^th^ PAM base	(50, 146–148)
xCas9(3.7)	Human (embryonic kidney cells^a^) Rice	Targets 5ʹ-NG-3ʹ, 5ʹ-NNG-3ʹ, 5ʹ-GAA-3ʹ, 5ʹ-GAT-3ʹ and 5ʹ-CAA-3ʹ PAMs Targets 5ʹ-NGG-3ʹ PAMs with higher efficiency than WT *Sp*Cas9 Much lower off-target activity than WT *Sp*Cas9 in human cells	(21, 149)
*Sp*Cas9-BE1, BE2, BE3, BE4	Human (embryonic kidney cells^a^) Rabbit (blastocysts) Sheep (one-cell stage embryos) *Xenopus laevis* (one-cell stage embryos) *Xenopus tropicalis* (one-cell stage embryos) Silkworm (embryonic cells)	300-900 human genetic diseases are potential targets for correction via base editing BE3 had the best editing yield of BE1, BE2 and BE3 BE4 showed higher C to T editing efficiencies, lower indel formation and higher product formation than BE3	(71, 98, 150–154)
*Sa*Cas9-BE3	Human (embryonic kidney cells^a^, osteosarcoma cells^a^)	Can target sites not accessible to *Sp*Cas9-BE3	(99, 155)
*Sa*KKH-BE3	Human (embryonic kidney cells,^a^ osteosarcoma cells^a^)	Targets 5ʹ-NNNRRT-3ʹ PAMs Higher efficiency of on-target editing than EQR-BE3 and VQR-BE3	(99, 155)
EQR-BE3	Human (embryonic kidney cells^a^, osteosarcoma cells^a^)	Targets 5ʹ-NGAG-3ʹ PAMs Less off-target activity than *Sa*BE3 and *Sa*KKH-BE3	(99)
VQR-BE3	Human (embryonic kidney cells,^a^ osteosarcoma cells^a^)	Targets 5ʹ-NGAN-3ʹ PAMs Less off-target activity than *Sa*BE3 and *Sa*KKH-BE3	(99, 155)
*Sp*Cas9 PE1, PE2, PE3	Human (embryonic kidney cells,^a^ osteosarcoma cells,^a^ leukemic bone marrow cells,^a^ HeLa cells,^a^ iPSCs^a^) Mouse (neuro-2a cells) Rice (protoplasts) Wheat (protoplasts)	75,000 pathogenic genetic variants diseases are potential targets for correction via prime editing Can perform insertions, deletions, all base conversions and combinations of these	(11, 156–160)

^a^ Application of CRISPR-Cas9 system only shown *in vitro*.

Whilst the studies explored in this review pave the way for making CRISPR-Cas9 an effective and safe tool, several hurdles spanning both science and society remain. Therefore, if maximum benefit is to be realized from this technology, future studies must widen their scope to consider the wider implications of their use and the longer-term impacts they might have on society and the natural world.
